# Artificial Intelligence in Digital Pathology: What Is the Future? *Part 2: An Investigation on the Insiders*

**DOI:** 10.3390/healthcare9101347

**Published:** 2021-10-11

**Authors:** Maria Rosaria Giovagnoli, Sara Ciucciarelli, Livia Castrichella, Daniele Giansanti

**Affiliations:** 1 Facoltà di Medicina e Psicologia, Università Sapienza Roma, Piazzale Aldo Moro, 00185 Rome, Italy; mr.giovagnoli.univ.sap@hotmail.com (M.R.G.); s.ciucciarelli.univ.sap@hotmail.com (S.C.); l.castrichella.univ.sap@hotmail.com (L.C.); 2 Centre Tisp, Istituto Superiore di Sanità, 00161 Rome, Italy

**Keywords:** e-health, medical devices, m-health, digital-pathology, picture archive and communication system, artificial intelligence, cytology, histology, diagnostic pathology

## Abstract

*Motivation:* This study deals with the introduction of artificial intelligence (AI) in digital pathology (DP). The study starts from the highlights of a companion paper. *Objective:* The aim was to investigate the consensus and acceptance of the insiders on this issue. *Procedure:* An electronic survey based on the standardized package Microsoft Forms (Microsoft, Redmond, WA, USA) was proposed to a sample of biomedical laboratory technicians (149 admitted in the study, 76 males, 73 females, mean age 44.2 years). *Results:* The survey showed no criticality. It highlighted (a) the good perception of the basic training on both groups, and (b) a uniformly low perceived knowledge of AI (as arisen from the graded questions). Expectations, perceived general impact, perceived changes in the *work-flow*, and worries clearly emerged in the study. *Conclusions:* The of AI in DP is an unstoppable process, as well as the increase of the digitalization in the *health domain*. Stakeholders must not look with suspicion towards AI, which can represent an important resource, but should invest in monitoring and consensus training initiatives based also on electronic surveys.

## 1. Introduction

In a complementary study [1] we dealt with the introduction of artificial intelligence (AI) in digital pathology (DP). This could lead to a *second revolution* in pathological diagnostics (starting from the *first revolution* determined by the introduction of DP techniques both in *eHealth* and *mHealth* [2,3]). Most AI applications [1] take place in diagnostic imaging. However, there are many important implications related to the introduction of AI. These implications involve *other disciplines* (not only connected to imaging) and *other activities*, from the *work-flow* to the training. In our study [1] we recalled the passages that led to the first revolution of diagnostic pathology, represented by DP. We dedicated particular attention to the critical issues, given that AI will rely heavily on it. In the same study, we highlighted the opportunities and the challenges of AI according to the most recent studies [4,5,6,7,8,9,10,11,12,13,14,15,16,17,18,19,20]. Some important development guidelines have been identified. The DP developments with AI have been identified [20]. AI shows in DP (A) the potentiality to access and correlate large amount of data, and (B) direct prospective in the world of diagnostics.

Regarding *A*, both radiological and pathology images are stored in the *picture archiving and communication systems* (PACs). Moreover, with the introduction of electronic health records (EHRs), systematic collections of patient health information have been made available. They include qualitative data, medical records, and laboratory and diagnostics information. AI, if applied to these large digital stores, could prove useful for epidemiological, clinical, and research studies.

Regarding *B*, two aspects are emerging:

- The development of the DP, due to the introduction of *whole-slide scanners* and the *progress* *of computer vision algorithms*, have significantly grown the usage of AI. It can perform tumor diagnosis, subtyping, grading, staging, and prognostic prediction
- The pathological diagnosis of the future could merge proteomics and genomics in the BIG-DATA.

The challenges to tackle and the evident opportunities of AI in DP were recently categorized in [19]. These challenges were therefore recalled in [1], starting from the grid identified in [19]. The following transversal issues to be considered in these challenges were introduced and discussed [1]:

1. Delay of digital cytology.
2. Greater complexity in the introduction of AI in digital cytology.
3. Focus on the DICOM WSI standard.
4. Attention to both eHealth and mHealth.
5. New training models must adapt to AI in DP.
6. Need for standardization actions.
7. Extensive acceptance surveys on professionals.
8. Need to focus on all the professionals involved.

All that is highlighted in the above cross-cutting issues is of strong scientific interest. These issues are basic to plan a routine introduction of AI in DP.

We intend with this study to concentrate on some of the points detected. 

We intend to propose a survey (*point 7*) focused on the professionals involved (*point 8*) to investigate the state of acceptance and the consensus on the introduction of AI in DP. Prior to this study, the experience reported in [21] focused on pathological diagnostics (*a single aspect of DP*), on a *single profession,* and proposed a non-validated and non-standardized questionnaire on the acceptance of AI in general. Despite limitations, several interesting findings were uncovered. Overall, respondents carried generally positive attitudes towards AI, excitement in AI as a diagnostic tool to facilitate improvements in *work-flow* efficiency, and quality assurance in pathology. Importantly, even within the most optimistic cohort, a significant number of respondents endorsed concerns about AI, including the potential for job displacement and replacement. Overall, around 80% of respondents predicted the introduction of AI technology in the pathology laboratory within the coming decade. The study focused on one single professional [21]; however, many other professionals are revolving around the introduction of AI in DP, ranging from the pathologist up to the biomedical laboratory technician. 

There are many other aspects to be taken into consideration besides the diagnostic aspects [21]. We must consider, for example [1,19,20], the peculiarity of digital cytology and of digital histology, omics (e.g., genomics and proteomics), integration with BIGDATA, integration with historical and clinical data of the patient, the search for slide labelling, quality control, the integration of DP with digital radiology, training, risk analysis, therapy, and prevention.

The goal of our study was to

▪ Propose an electronic survey (based on a standardized software package) dedicated to the introduction of AI in DP, considering both the opportunities of AI in their entirety [1,19,20] (not limited to pathological diagnostics) and the involved professionals.
▪ Submit it electronically to a first sample of insiders.
▪ Analyze the outcome.

## 2. Materials and Methods

In line with the aim of the study, we decided to propose a survey to investigate the acceptance and the consensus of the insiders. Preliminarily, we addressed the aspects of privacy and data security. The questionnaire was checked for the compliance to the European GDPR 679/2016 and the Italian Decree 101/2018, as required by the Data Protection Offices. The questionnaire was planned as anonymous. The topic did not concern clinical trials on humans, but only opinions and expressions of their thoughts. In consideration of this, it was not considered necessary to proceed with the formal approval procedures from the Institutional Review Board (see footnote at the end). The standard Microsoft Forms package (Microsoft Forms, Redmond, WA, USA) was chosen.

This package is also available with a free Microsoft account (live, outlook, or hotmail, for example), but in this case, it has important limitations (for example, the maximum number of participants is limited to 200). The data acquired by means of Microsoft Forms represent a public register from a legal point of view. Therefore, data need to be strongly protected by means of a strong cybersecurity approach. This is not feasible using only a free Microsoft account.

Companies that have centrally installed the Microsoft 365 App Business Premium suite have Microsoft Forms available to their users with greater potential than the free version (for example, the maximum limit of participants is raised to 50,000). All users can have access through their own domain account guaranteed by the corporate cybersecurity standards (which must comply with the international regulations in force) supported by network and system security tools and policies managed by the company. Specific checks are possible on the IPs (registering, for example, the duplicate access for further data-process). Data are therefore protected by the corporate cybersecurity systems, guaranteeing (at least from the system point of view) the inviolability of the data. In consideration of this, we have decided to use the software Microsoft Forms, provided through the Microsoft 365 Business Premium suite, to design an electronic survey. It is the tool recommended by the company’s DPO. It should be noted that if a tool other than those available in this suite (e.g., Google forms or Survey Monkey) had been used, the DPO would have requested a specific report and a cybersecurity audit. The authorization to use it would not have been guaranteed. The use of both an internally recommended tool (respecting the cybersecurity) and the plan to submit the electronic survey (eS) anonymously simplified the authorization process. However, we decided to maintain the database as a register, respecting the security criteria identified by the company rules in accordance with the law. The procedure used in the design and submission of the survey adhered to the *SURGE Checklist* [22].

We decided to submit the survey to the key professionals and therefore disseminated it through social media, such as Facebook, LinkedIn, Twitter, Instagram, WhatsApp, Association Sites, and in general, following a *peer-to-peer* dissemination. We submitted the survey to biomedical laboratory technicians during their course of study (*BLT-DCS*) and after the course of the study (*BLT-ACS*). The interactive survey is available in [23]. A print can also be found in [24].

**Figure 1:**
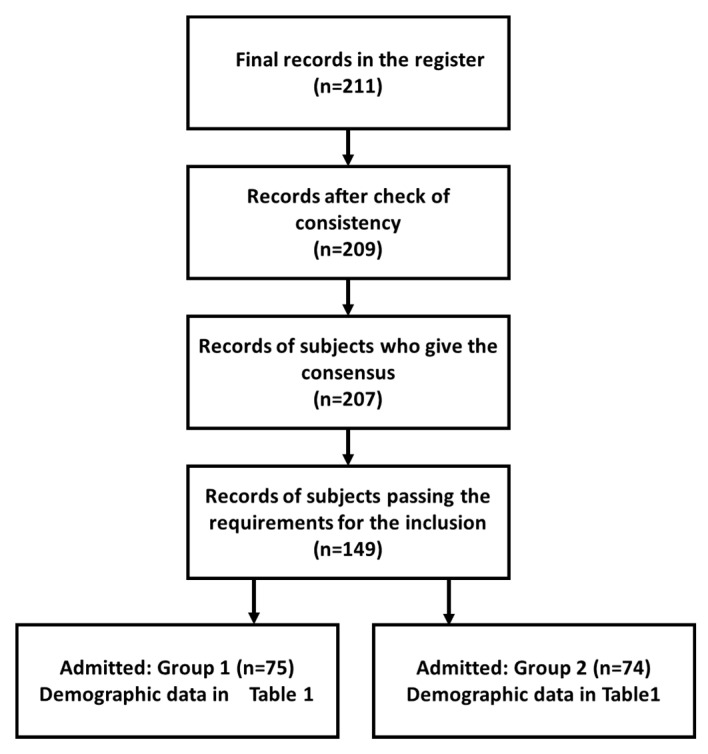
The CONSORT diagram.

Two questions (N.2 and N.3) stratify by age and sex [23,24]. Two initial questions (N.4 and N.5) categorize the sample on the basis of the training background. In consideration of the objective of this study and the survey, we also managed the survey as a virtual focus group, with careful considerations to the consensus issues related to all the aspects of the introduction of AI in DP [1,19,20]. We started from the training up to the relationships and integration with omics, BIG-DATA, and digital radiology. The methodological approach primarily involves submitting both to *BLT-DCS* and *BLT-ACS* surveys. Figure 1 shows the CONSORT diagram. The final records were 211 in number. Two records were excluded because the answers to the open questions were not coherent. 

The subjects passing the requirements for the inclusion according to the selection criteria (*BLT-DCS* or *BLT-ACS*) were 149 (Table 1).

The quantitative variables depended on subjective answers based on qualitative perceptions (see for example in the following the graded questions or the modules in the Likert scale). The survey used *open question, choice question, multiple choice questions, Likert questions,* and *graded questions*.

We established a six-level psychometric scale for the *Likert scale* and the *graded questions*. It was possible therefore to assign a minimum score of one and a maximum of six with a *theoretical mean value* (TMV) of 3.5. We can refer to the TMV for comparison in the analysis of the answers. An average value of the answers below TMV indicates a more negative than positive response. An average value above TMV indicates a more positive than negative response.

The trend of each one of these variables, estimated by an average value, can move in both the two directions, toward the higher score of 6 or toward the lower score of 1, suggesting for a two-tailed test. For the variables related to the *multiple-choice* questions, we planned a frequency analysis.

For the verification of data normality, we used the Shapiro–Wilk test that is preferable for small samples such as ours.

We applied Student’s *t*-test (with a *p*-value <0.01 for the significance of the difference), when comparing the values between the two groups.

We applied the χ^2^ test (with a *p*-value <0.01 for the significance) in the frequency analysis. The software SPSS Statistics version V.24 was used in the study.

The Cohen’s d effect size was estimated to be 0.498. Samples with N > 60 were estimated suitable to the study.

We established a six-level psychometric scale in the graded questions and in the Likert scale.

The survey was proposed from 1 June 2021 until 23 August 2021.

## 3. Results

Table 2 shows the answers to the graded *questions*. Both questions Q6 and Q7, not focused directly on AI, received an average response value above the TMV threshold.

However, Q6 showed a significantly higher value in the student group (*p*-value < 0.01), while Q7 showed a consistent value between the two groups (*p*-value = 0.134 >> 0.01).

The responses related to AI, Q8–10, showed a value below the current TMV threshold in the two groups (*p*-value < 0.01).

Table 3 and Table 4 highlight the outcomes for the two Likert scales in detail. In the first Likert scale (Table 3), *imaging* (cytological and histological) received the highest score for the two groups, followed by applications in *omics and quality control*. Table 4 shows the significant highest values for the first group in the second Likert scale dedicated to other sectors of applications.

The *multiple-choice questions* are useful for obtaining strategic information, for example, for scientific societies or consensus activities. We decided to proceed as follows, in consideration of the peculiarity of these modules. We analyzed the two samples joined into one sample and performed a statistical approach based on a frequency analysis, using the test described in the methods.

For question Q13 “*I think artificial intelligence in my field*”, the two most popular statements were “*It will be useful but complementary*” number of votes = *83* and “*It will not catch on*” number of votes = *78* (*p*-value = 0.008).

For question Q14 “*How can I be of use to AI in my filed*”, the two most popular statements were “*In performance monitoring”* number of votes = *90* and “*As an operational manager of its use*” number of votes = *81* (*p*-value = 0.008).

For question Q15 “*How will AI help me*”, the two most popular statements were “*Increased automatism*” number of votes = *79* and “*Reduction of physical fatigue*” number of votes = *61* (*p*-value = 0.009).

## 4. Discussion and Conclusions

The use of AI is increasingly spreading in many medical sectors.

A particularly important area for applications is that of images. A simple search on PubMed with the key

*(artificial intelligence [Title/Abstract]) AND (image [Title/Abstract])*

shows 2290 results as of 23 August 2021 (907 in 2021).

*This justifies the need of focusing on studies of acceptance, in consideration of both the interest of the scholars and the possible opportunities in the clinical routine*.

Some studies are also demonstrating the importance of AI tools, not only in imaging, but also in other applications where *data mining from large volumes of data must be applied*.

For example, the study reported in [25] showed how AI is useful for determining cardiovascular risk in athletes through *data mining of distributed databases*.

The COVID-19 pandemic has also highlighted *the broad-spectrum potential of AI*. In a recent review [26], for example, relevant papers were selected that address the adoption of artificial intelligence and new technologies in the management of pandemics and communicable diseases such as SARS-CoV-2.

These studies focused on environmental measures; acquisition and sharing of knowledge in the general population and among clinicians; development and management of drugs and vaccines; remote psychological support of patients; remote monitoring, diagnosis, and follow-up; and maximization and rationalization of human and material resources in the hospital environment. The study described in [27] showed that *AI-based scores with a purely data-driven selection of features* are feasible and effective for the prediction of mortality among patients with COVID-19 pneumonia.

*The three illustrated potentials [25,26,27] are also important in DP. In fact, in DP, the need for categorizing images merges with the need to make decisions and/or deduce approaches through actions on large databases and data sets or with other needs not based on medical images [1,19,20]. The implications are multifaceted. It is necessary to carry out direct studies on the opinion of insiders in view of the introduction of the clinical routine of AI*.

*Therefore, the need and the justification of studies such as ours that tackle the introduction of AI focusing on acceptance and with a broad approach clearly emerges from these articles [25,26,27]*.

Very few studies have begun to address the insiders’ opinion on the introduction of AI in DP. By searching in PubMed with the key

*((digital pathology [Title/Abstract]) AND (artificial intelligence [Title/Abstract])) AND (survey [Title/Abstract])-even with alternative terms to the survey-*

we found as of 23 August 2021 only two studies based on non-validated and non-standardized questionnaires.

The first study [28] was conducted at a scientific meeting (the 14th Banff Conference). Since the meeting, a survey with international participation of mostly pathologists (81%) was conducted, showing that whole slide imaging is available at the majority of centers (71%), but that artificial intelligence (AI)/machine learning was only used in ≈12% of centers, with a wide variety of programs/algorithms employed.

The second study [29] reports the results of the Japanese questionnaire survey conducted in 2008–2009 on telepathology and virtual slide. Moreover, in addition to the questionnaire, the effectiveness of an experimental automatic pathology diagnostic aid system using computer artificial intelligence was investigated by checking its rate of correct diagnosis for given prostate carcinoma digital images.

This demonstrates the importance of focusing on wide-ranging survey studies in this field. From this research, it clearly emerges that specific studies, such as ours based on wide range questionnaires, have not been addressed until now. In fact, in the literature, there are currently only studies that deal with the topic only partially or secondarily [28,29].

This study was necessary to prepare a first survey dedicated to the acceptance of AI in DP focused on the insiders [1]. We submitted the survey on the professionals involved in the field. Many professionals are involved in the introduction of AI in DP, ranging from the bioengineer to the pathologist up to the biomedical laboratory technician. There is also no doubt that AI could represent a serious opportunity for the DP laboratories [5,6,7,8,9,10,11,12,13,14,15,16,17,18]. It is, however, the time to investigate the full introduction in the routine. The proposed study, for example, can be useful in view of consensus studies on the introduction of methods based on AI in DP in routine practices [1,19]. We have proposed a survey focused on these professionals that is, in an automatic manner, capable of electronically collecting their opinion and works as a structured virtual focus group.

The intent of this study was to carry out a first submission and to verify any criticalities in view of a wider use. There were no critical issues and the submission made it possible to collect information on a first sample of biomedical laboratory technicians in the training phase and subsequent phase.

A good perception of the basic training on both groups (albeit with a different score) and a uniformly low perceived knowledge of the use of AI emerged from the graded questions.

The *two Likert scales* made it possible to identify in a structured way, for the two groups, the wishes related to the use of AI in the medical field.

The *multiple-choice* questions, evaluated for the whole combined sample, allowed us to evaluate the perceived impact of AI in one’s sector, the expectations towards AI, and the operational role towards AI. From a general point of view, the study presents three added values.

The *first added* value is [23,24] represented by the electronic tool with a wide range of aspects related to the use of AI in DP, having a direct impact on the *work-flow* and *job description* of the insiders.

The *second added* value is a contribution directed to respond to the need to tackle the challenges of the introduction of AI in DP. This product (after minimal changes) could be used by scientific and/or professional societies to monitor the evolution of the topic.

The *third added* value is represented by the outcome with reference to the two groups of *DCS* and *ACS* (promptly useful for the stakeholders).

From a general point of view, this article supports the initiatives that aim to facilitate the introduction of AI in a structured manner in DP. Future developments of the study foresee the enlargement of the submission to other professionals and a standardization for the scientific societies.

## 5. Limitations

This study represents a first step to investigate the acceptance and consensus on AI of insiders in the various applications and implications of DP. It was applied to a first professional and a first group of subjects. Future developments will have to include a broader submission involving other professionals, together with a review action by the scientific societies, in order to improve acceptance by the parties involved.

## Figures and Tables

**Table 1:** Characteristics of the admitted to the study the DCS and the ACS.

Submission	Participants	Males/Females	Min Age/Max Age	Mean Age
*Biomedical laboratory technicians under the course of the study* *(BLT-DCS)*	75	39/36	21/36	25.3
*Biomedical laboratory technicians after the course of the study* (*BLT*-*ACS)*	74	37/37	25/59	41.8

**Table 2:** Answers for the graded questions.

Feature	Rating DCS	Rating ACS	*p*-Value
**Q6:** **Degree of knowledge in computer science**	4.8	4.9	0.009
**Q7:** **Degree of knowledge of biomedical technologies**	4.7	4.7	0.134
**Q9:** **Degree of knowledge of AI (in general)**	3.3	3.1	0.009
**Q10:** **Degree of knowledge of AI (in biomedical sector)**	3.4	3.2	0.009
**Q11:** **Degree of direct knowledge of technologies and applications of AI (in biomedical sector)**	1.8	1.3	0.008

**Table 3:** Detailed answers in the Likert scale to the question of “In which specific sectors of biomedical diagnostics do you think the introduction of artificial intelligence is most promising?”.

Feature	Rating DCS	Rating ACS	*p*-Value
**Digital cytology**	4.9	4.5	0.008
**Digital histology**	4.8	4.4	0.009
**Omics (e.g., genomics and proteomics)**	4.6	4.3	0.008
**Integration with BIG-DATA**	3.9	3.7	0.008
**Integration with historical and clinical data of the patient**	4.1	3.8	0.009
**Search for slide labeling**	3.9	3.6	0.009
**Quality control**	4.1	3.8	0.009
**Integration of DP with digital radiology**	4.2	3.9	0.009
**Quality control**	4.5	4.2	0.008
**Integration with the virtual medical record**	3.9	3.7	0.008
**Training**	3.9	3.6	0.008

**Table 4:** Detailed answers in the Likert scale to the question of “In which more general sectors do you think artificial intelligence is useful?”.

Feature	Rating DCS	Rating ACS	*p*-Value
**Risk analysis**	4.3	3.7	0.008
**Therapy**	4.4	3.8	0.008
**Prevention**	3.9	3.6	0.009

## Data Availability

Not applicable.
